# Editorial: Ruminant mastitis: A 360° view

**DOI:** 10.3389/fvets.2022.1055323

**Published:** 2022-10-27

**Authors:** Federica Riva, Alejandra A. Latorre, Paolo Moroni

**Affiliations:** ^1^Department of Veterinary Medicine and Animal Sciences (DIVAS), University of Milan, Milan, Italy; ^2^Departamento de Patología y Medicina Preventiva, Facultad de Ciencias Veterinarias, Universidad de Concepción, Chillán, Chile; ^3^Quality Milk Production Services, Animal Health Diagnostic Center, Cornell University, Ithaca, NY, United States

**Keywords:** mastitis, intra-mammary infection, dairy ruminants, antibiotics, mastitis pathogens

In dairy ruminants, mastitis represents one of the most serious health issues which can contribute to the reduction of milk production, high veterinary care costs, increased use of antibiotics, and animal culling, all of which may account for severe economic losses. Mastitis consists of the inflammation of the udder and is mainly caused by contagious and/or environmental microorganisms leading to overt clinical or sub-clinical cases. Management of mastitis in dairy ruminants faces various challenges: the resilience of high-yielding animals, poor efficacy of therapies and prevention (antibiotic resistance, dubious efficacy of vaccines), quality of milk, reduced availability of food and water, climate change, and difficulties in early diagnosis. These issues could provoke the interest of researchers and practitioners to suggest more affordable and effective control measures.

This Research Topic is comprised of 20 articles. These include review articles, original research, and reports concerning the biology of mastitis pathogens, a comparison of several diagnostic tools, diverse prevention strategies, alternative treatments to antibiotics, and the prevalence of mastitis-causing agents.

The study of pathogens, pathogenesis, and host-pathogen interaction could be helpful in the definition of new prevention or treatment protocols for combating mastitis. Non-Aureus Staphylococci (NAS) involves several species and strains that have become the most frequently isolated bacteria in milk. Non-Aureus Staphylococci are associated with mastitis, with variable severity of cases, and some species are considered commensal in the mammary gland. These aspects prompted the scientific community to investigate the biology, pathogenicity, epidemiology, and antibiotic resistance of bacteria member of the NAS group (De Buck et al.). In emerging countries, different genetic strains of *Staphylococcus aureus* (SA) have been found in milk samples. Some of these strains are known to be adapted to dairy ruminant species but a highly diffuse one is a human-adapted strain. As all SA strains carry different antibiotic resistance genes, their isolation from milk poses important zoonotic concerns (Ndahetuye et al.). In China *Helcococcus ovis*, a mastitis-causing agent in sheep, was isolated on a dairy cow farm. Four genetic strains were identified, all susceptible to the majority of antibiotics. *Helcococcus ovis* caused mild mastitis in a murine model when it was the only bacteria causing infection, but it caused severe mastitis when co-infecting the murine mammary gland with *Trueperella pyogenes* (Liu et al.). The role of co-infection in mastitis has been poorly investigated but it reveals interesting aspects for the management and treatment of mastitis. For example, the role of a viral infection such as the Bovine Leukemia Virus (BLV), can impair the defenses in the mammary gland predisposing it to secondary bacterial infections (da Souza Lima et al.). The knowledge of the pathogenesis of each agent could help in the management of this pathological condition for the udder. The mechanism by which SA infection impairs the milk protein synthesis by mammary epithelial cells has been investigated by Chen et al. demonstrating the involvement of mTORC1, STAT5, NF-kB, and two amino acid transporters (SLC1A3 and SLC7A5). Another aspect is the debated capability of some mastitis agents to develop biofilms that could explain their persistence in the udder, resistance to antibiotic treatments, and the presence of some chronic infections (Pedersen et al.). Moreover, in small ruminants such as high milk production goats, genetics may play a role in certain histological features that increase the permeability of skin to milk (“Weeping Teats” or WT), but limited information was available regarding an increased risk of mastitis on animals with WT. Gazzola et al. demonstrated a positive association between high SCC and bacteria in milk from goats suffering from WT, but these variables were not associated with the WT condition.

Dairy ruminant farming should try to develop early diagnostic tools and mastitis prevention strategies. Mastitis can occur as sub-clinical, mild clinical, or severe clinical pathological conditions. Depending on its appearance, the diagnosis could encounter difficulties. Somatic cell count (SCC) is the most widely used indicator of intra-mammary infection (IMI), but depending on the number of days in milk (DIM), cow breed, or other factors, the SCC value is sometimes inconclusive. The definition of the differential SCC (DSCC) has been proposed as a further indicator of IMI. The automated DSCC has become available recently with two instruments. Halasa and Kirkeby, and Alhussien et al. discussed insights about the use of the DSCC. They suggest that the collective use of SCC, DSCC, and evaluation of phagocyte activity is certainly helpful in the discrimination of different mastitis forms. They discussed the advantages and disadvantages of the two instruments, one for field investigations and the other for laboratory investigations. Another study proposed chromogenic media for the rapid identification of bacteria causing subclinical mastitis (SCM). Garcia et al. demonstrated the capability of this chromogenic medium to identify the main Gram-positive bacteria responsible for subclinical mastitis, with the exception of *S. aureus*.

Despite the improvement of the technologies for the management of dairy herds and milking, combined with the amelioration of veterinary interventions, IMI still remains an important issue in the dairy industry. For this reason, measures of prevention play a pivotal role in the reduction of the risk of mastitis. As documented by Zigo et al., given the multifactorial nature of the pathological condition of mastitis, the prevention strategies should relate to the control of SCC, correct diet, proper housing and management, milking systems, drying strategies, and eventually, immunization protocols. Research in the mastitis prevention field is very dynamic and new tools are often being proposed. Kabera et al. after a comparison between selective dry cow antimicrobial treatment (SD) and blanket dry cow treatment (BD), suggested applying SD in regard to antibiotic use reduction. Bedding choice can influence mammary gland health. Fréchette et al. highlight the risk of *K. pneumoniae* mastitis when using recycled manure solids bedding. The efficacy of immunizing dairy ruminants against mastitis is debated, but efficacious vaccines could represent a strong defense for combating IMI. Tassi et al. describe the development of an efficacious intramammary vaccine against *Mannheimia haemolytica*, based on the activation of innate immune responses. Effective vaccination against mastitis is not available and the causes of the failure of this type of prevention still need to be investigated. New control strategies such as immunomodulation may be helpful for the future fight against mastitis.

The treatment of mastitis is based mainly on antibiotics but the widespread increase of antibiotic-resistant bacteria poses several concerns about their use and stimulates the search for new therapeutic strategies. As stated by Ruegg, there are very few studies that investigate the real efficacy of antibiotic treatment. In order to develop efficacious therapeutic protocols for the treatment of mastitis, it is important to evaluate several aspects such as the pathogen involved, activity spectrum, antibiotic resistance, and animal factors (parity, stage of lactation, previous mammary infections). Non-steroidal anti-inflammatory drugs (NSAID) alone have been proposed by Krömker et al. as an alternative treatment to antibiotics, but only in case of mild or moderate clinical mastitis AMPK/NrF2/Nf-kB signaling pathway has been suggested as a potential target for the treatment of *S. aureus* bovine mastitis by Arbab et al.

Mastitis control and prevention are based on continuous and accurate monitoring of the bacterial species prevalence and evaluation of their antibiotic resistance. In our collection, we included recent data about the prevalence of mastitis pathogens in Northwest Pakistan (Ali et al.), Ontario, Canada (Acharya et al.), and the prevalence of *Klebsiella* spp. in China (Liu et al.). The distribution of mastitis pathogens around the world suggests the need for the application of differential strategy programs to control the disease in different countries.

## Author contributions

All authors listed have made a substantial, direct, and intellectual contribution to the work and approved it for publication.

## Conflict of interest

The authors declare that the research was conducted in the absence of any commercial or financial relationships that could be construed as a potential conflict of interest.

## Publisher's note

All claims expressed in this article are solely those of the authors and do not necessarily represent those of their affiliated organizations, or those of the publisher, the editors and the reviewers. Any product that may be evaluated in this article, or claim that may be made by its manufacturer, is not guaranteed or endorsed by the publisher.

